# Design and implementation of a phasor measurement unit using a new measurement technique

**DOI:** 10.1038/s41598-026-49889-y

**Published:** 2026-05-05

**Authors:** Samah A. Mohamed, Hala M. Abdel Mageed, Osama M. Arafa, Mohamed H. Merzban, Magdy B. Eteiba, Ahmed S. Haiba

**Affiliations:** 1 Electrical Department, Faculty of Engineering, Fayoum University, Fayoum, Egypt; 2 Electrical Power, Energy and High Voltage Metrology Laboratory, National Institute of Standards (NIS), Giza, Egypt; 3 Power Electronics and Energy Conversion Department, Electronics Research Institute, Cairo, Egypt

**Keywords:** Global positioning system (GPS), Phasor measurement unit (PMU), Electrical power system (EPS), Power system state, Renewable energy, Synchro phasors, Energy science and technology, Engineering

## Abstract

Power system dynamics are being significantly impacted by the widespread use of renewable energy sources, and modern electrical networks are growing increasingly complex. This trend highlights the need for new monitoring technologies to ensure the continuous operation and operational efficacy of the electrical network. Similar to Phasor Measurement Units (PMUs), a synchronized monitoring system provides a solid basis for the intricate power system and can be a helpful tool in challenging situations. This paper presents the design and realization of a (PMU) based on a new measuring technique pivoted on Sliding Fourier Transform Phase-locked loop (SFT-PLL). This technique enhances the accuracy and dynamic performance of phasor estimation in modern power systems. The PMU prototype was first validated through simulation and then implemented as a hardware prototype. the complete hardware architecture of the PMU has been realized using analog signal conditioning circuits, a high-resolution analog-to-digital conversion stage, a real-time processing platform based on Texas Instruments Delfino F28379D board, and Global Positioning System (GPS) Module. Experimental results demonstrate that the proposed PMU achieves higher accuracy in calculating magnitude, phase angle, and frequency, while maintaining fast dynamic response during severe grid disturbances. These outcomes confirm that the new measurement technique significantly improves phasor estimation performance and enhances the reliability of real-time monitoring in modern power systems.

## Introduction

Phasor Measurement Units (PMUs) have become extremely important in today’s power systems. They are used in many tasks, such as load shedding, managing the distribution grid, monitoring system conditions, and estimating the power system state. This shows how valuable PMUs are in modern electrical networks. One of the main requirements of a smart grid is having very accurate, time-synchronized measurements and PMUs make this possible. With their precise data, operators can get a clearer picture of what is happening in the system and even predict what might happen next. If changes in voltage or phase angle start to indicate a problem, PMUs help detect these issues quickly so that disturbances can be addressed before they spread^1^. Phasor measurement units (PMUs) can be used for control room applications to measure electrical signals like voltage, current, and frequency with a common time reference that is GPS time-stamped, where a synchro phasor is both the magnitude and the angle of a cosine signal of electrical current or voltage that can be measured in an absolute point in time^2^. In the literature, a lot of algorithms are presented to estimate the phasor and improving PMU performances using various algorithms such as Sliding Discrete Fourier Transform (SDFT)^3^, all-phase fast Fourier transform (apFFT)^4^, The Interpolated Discrete Fourier Transform (IpDFT)^5^, Taylor Fourier Multifrequency Model^6^ and Clarke Transformation Based DFT algorithms^7^. Taylor-Kalman filters^8^, Adaptive Cascaded filters^9^, Polynomial Phase-Locked-Loop Taylor–Fourier Filters^10^, a double suboptimal-scaling factor adaptive strong tracking Kalman filter (DSTKF)^11^ and many others. However, there are certain studies that concentrate on using these kinds of tools to implement the PMU. The paper^12^ introduced a simple, low-cost PMU design. The whole system data acquisition, processing, and communication was built around one inexpensive microcontroller, a basic GPS receiver was used for synchronization, and signal-processing techniques were applied to match the sampling with UTC time and for estimating the synchro phasor, the design used the Interpolated DFT (IDFT) method. The paper^13^ introduced a low-cost PMU prototype called PhasorsCatcher, designed to measure frequency and rate of change of frequency in power systems on the three Arduino modules (the Arduino Mega2560). in Paper^14^, a low-cost PMU designed for distribution grids is presented and evaluated for its accuracy according to the IEEE C37.118.1–2011 standard and for calculating the system frequency, a zero-crossing detection algorithm was applied, the phasor measurements, on the other hand, were computed using the Discrete Fourier Transform (DFT), it was built using several modular components running on devices such as the Raspberry Pi or Beagle Bone Black. A PMU built on a low-cost microcontroller, which combines data acquisition, processing, and communication within a single unit, is presented in^15^. The study evaluates its performance under steady-state conditions with frequency deviations and harmonic distortions. In^16^, another low-cost PMU prototype was developed using a Digital Signal Processor (DSP), designed to comply with the IEEE C37.118 standards^17^, and tested for frequency estimation across different operating scenarios. the paper^18^ introduced a fully open-source PMU platform covering both hardware and software that is low-cost, high-performance, and expandable. The system incorporated two processing modules (a TI TMS320c5517 DSP and an Octavo OSD3358 processor), dual 64-GB local databases, a GPS module, a 5G modem interface, and analog/signal conditioning circuits for three-phase voltage and current inputs. The prototype has already been deployed and tested in several medium and low voltage substations in Cyprus, Spain, and Italy.

Other efforts toward developing low-cost PMUs include the work titled *“Design of an Inexpensive Residential Phasor Measurement Unit”* presented in^19^, along with the MSc theses of Onisokonikumen Valiant Sampson^20^ and Debashish Mohapatra^21^. Several studies have reported practical PMU implementations based on open-architecture hardware and software. Notable examples include Mohapatra’s design^21^ and the Open PMU project^22^, which provides a comprehensive description of a fully functional PMU developed using open-source hardware and software, accompanied by the complete software code. The Open PMU project also documents the implementation of its first prototype (Open PMU V1) using National Instruments (NI) hardware and LabVIEW software^23^. Detailed assembly instructions and software installation guidelines are provided, making it a valuable platform for PMU development^24^. Nonetheless, its reliance on proprietary NI hardware significantly increases the cost, particularly when the required software licenses are not already available.

Recent studies on synchrophasor estimation have highlighted the importance of integrated compliance evaluation. Kumar (2025) proposed an M-class estimator based on an enhanced band-pass FIR filter and validated its performance under comprehensive tests, including both steady-state and dynamic conditions^25^ and Paper^26^ has proposed low-cost PMU implementations capable of operating under off-nominal frequency and decaying DC conditions. An enhanced estimation framework combining harmonic filtering, frequency compensation, sample value adjustment, and modified FCDFT was validated according to IEEE C37.118.1a under both steady-state and dynamic tests. The reported results demonstrated compliance with TVE and FE limits and confirmed real-time feasibility using an embedded ESP32 platform. Most synchrophasor estimation methods are based on the Discrete Fourier Transform (DFT) due to its simplicity and efficiency. However, DFT suffers from spectral leakage, sensitivity to frequency deviations, and reduced accuracy under dynamic or distorted conditions. To overcome these limitations, Phase-Locked Loop (PLL) approaches, especially Synchronous Reference Frame PLL (SRF-PLL), have been proposed in^27^. In this method, three-phase signals are transformed into the dq reference frame and appropriate filtering techniques, such as Finite Impulse Response (FIR) filters to extract the positive-sequence component while improving noise immunity. The SRF-PLL then estimates amplitude, phase, and system frequency in real time, and additional processing can compute ROCOF to enhance dynamic performance and meet IEEE C37.118 requirements.

This paper presents the design and implementation of a Phasor Measurement Unit (PMU) using a modern measurement approach called the Sliding Fourier Transform – Phase Locked Loop (SFT-PLL), which improves both the accuracy and dynamic performance of phasor estimation in modern power systems. The proposed method incorporates an enhanced digital signal processing algorithm that can accurately extract voltage and current phasors even in the presence of noise or signal distortion, outperforming traditional techniques such as the Discrete Fourier Transform (DFT). The PMU prototype was first validated through simulations and then implemented in hardware. The SFT-PLL algorithm was developed in MATLAB Simulink, and the complete hardware architecture includes analog signal conditioning circuits by using three voltage and current sensors manufactured by PEMODULE, capable of measuring up to 600 Volts and up to 5 Amperes respectively, a high-resolution analog-to-digital conversion stage, and a real-time processing platform based on the Texas Instruments Delfino F28379D board, complemented by a GPS module.

### Research gaps and motivation

Although SFT-PLL-based techniques have been previously introduced in the literature, most existing studies primarily focus on the algorithmic structure. Limited attention has been given to its integration within a complete three-phase PMU architecture that simultaneously processes both voltage and current signals. Moreover, previous works often evaluate PLL performance in isolation without conducting comprehensive validation under steady-state and abnormal grid operating conditions. Therefore, the motivation of this work is to bridge the gap between theoretical SFT-PLL formulations and practical PMU realization by:

- Integrating SFT-PLL into a complete three-phase PMU framework.
- Implementing synchronized estimation of three-phase voltage and three-phase current.
- Validating performance under steady-state and abnormal operating conditions.
- Evaluating accuracy using standard performance indices.

Accordingly, the contribution of this study lies in practical system-level implementation and comprehensive validation, rather than in proposing a new synchronization algorithm.

## Proposed phasor measurement unit (PMU)

**Fig. 1:**
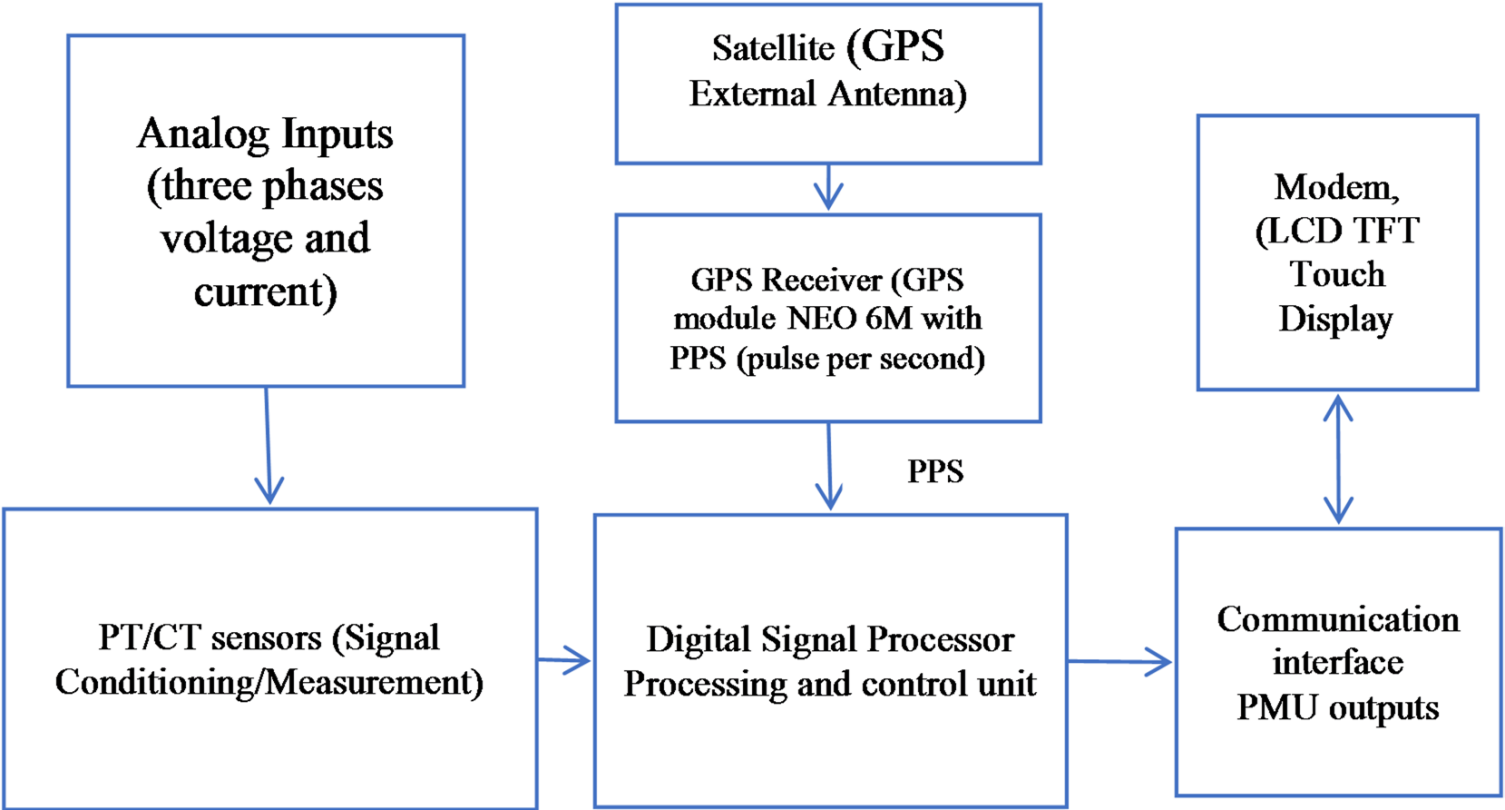
Block diagram of the proposed PMU.

The proposed Phasor Measurement Unit adopts the conventional structure commonly found in commercial devices. As illustrated in Fig. 1, it is composed of three primary functional units. The block diagram outlines the three main stages involved in the design and implementation of the PMU: the signal conditioning and measurement stage, the processing and control unit, and the PMU output and communication stage.

The first unit is the Signal Conditioning and Measurement stage, where the three-phase voltage and current signals are scaled, filtered, and converted into forms suitable for digital processing. This stage includes isolation circuits, analog filters, and the ADC interface responsible for precise sampling. The second unit is the Processing and Control stage, which hosts the core phasor estimation algorithm. Here, the sampled data are processed using digital signal processing techniques to compute the synchro phasors, frequency, and rate of change of frequency (ROCOF). This unit also manages time synchronization, data validation, and system control tasks. The final unit is the PMU Output and Communication stage, which formats the computed measurements according to standard communication protocols and transmits them to external systems such as PDCs, SCADA, or monitoring platforms. This stage ensures reliable data exchange through serial, Ethernet, or wireless interfaces depending on the required specific design requirements.

### Signal conditioning and measurement stage

**Fig. 2:**
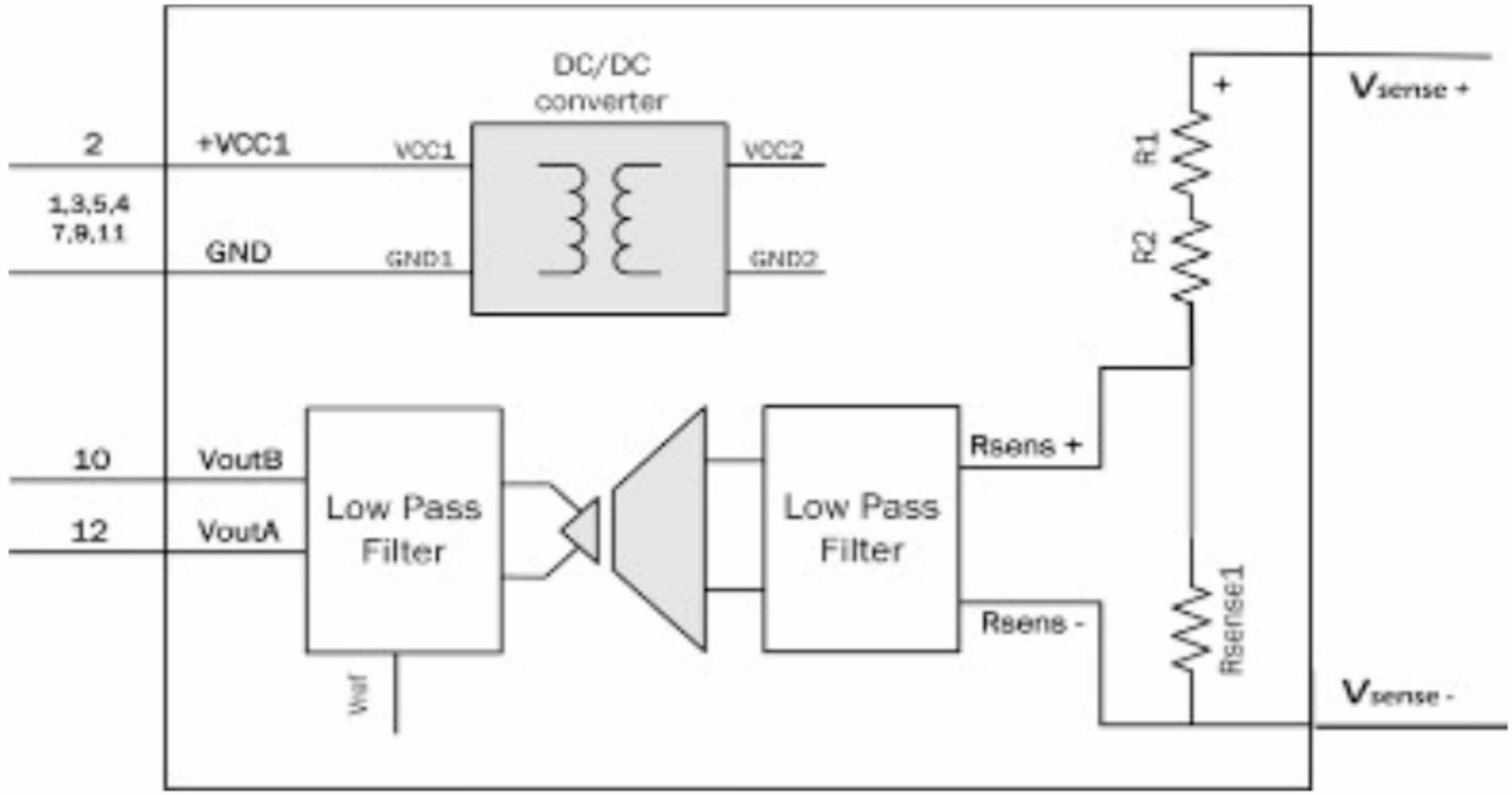
Block diagram of the voltage sensor.

**Fig. 3:**
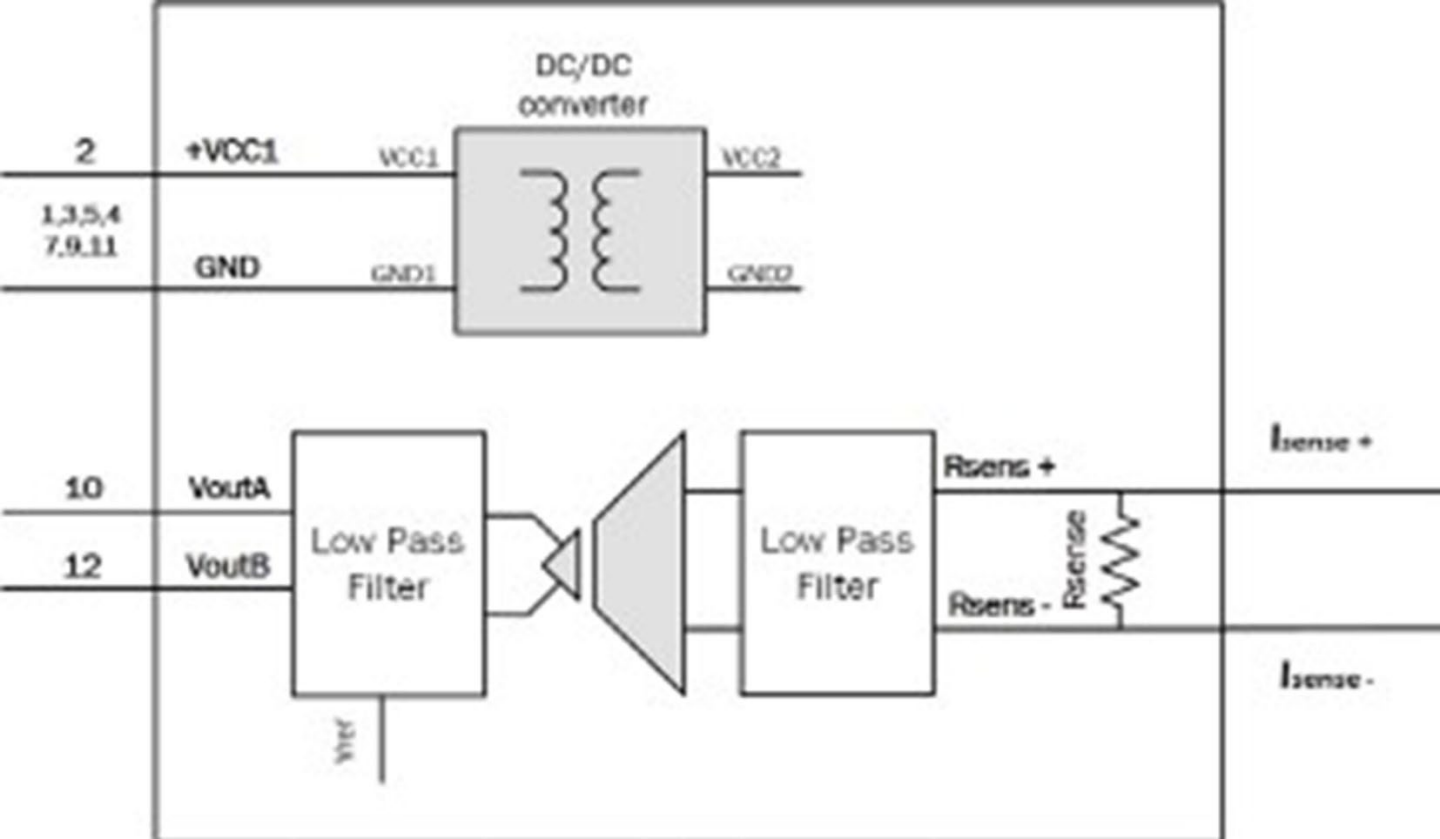
Block diagram of the current sensor.

Converts high-voltage and current signals into measurable low-level analog signals suitable for the analog-to-digital converter (ADC). It includes isolation transformers, potential transformers (PTs), current transformers (CTs), and low-pass filters that Represented by three voltage sensors and three current sensors manufactured by PEMODUEL, capable of measuring up to 600 Volts and up to 5 Amperes, respectively. These sensors are used to measure analog voltage and current values and subsequently convert them into analog voltage outputs with a low range of (± 5 V) on the BNC output (for bipolar use) or solely unipolar values ranging from 0 to 3 V (0–3 V) on the VIDC output (For DSP use). Figure 2 shows the block diagram of voltage sensor as similar Fig. 3 shows the block diagram of current sensor and Table 1 shows the calibrated sense conversion equations of three Voltage and current sensors.

**Table 1:** The calibrated sense conversion equations of three voltage and current sensors.

sensors	Equation of current sensors	Equation of voltage sensors
Sensor (1)	I_SENS_ =( VIDC − 2.0270)*3.422	V_SENS_ = (VIDC − 2.0270) *554.9
Sensor (2)	I_SENS_ =( VIDC − 2.0315)*3.465	V_SENS_ = (VIDC − 2.0315) * 556.9
Sensor (2)	I_SENS_ =(VIDC − 2.0420)*3.433	V_SENS_ = (VIDC − 2.0420) * 552

### Processing and control unit

**Fig. 4:**
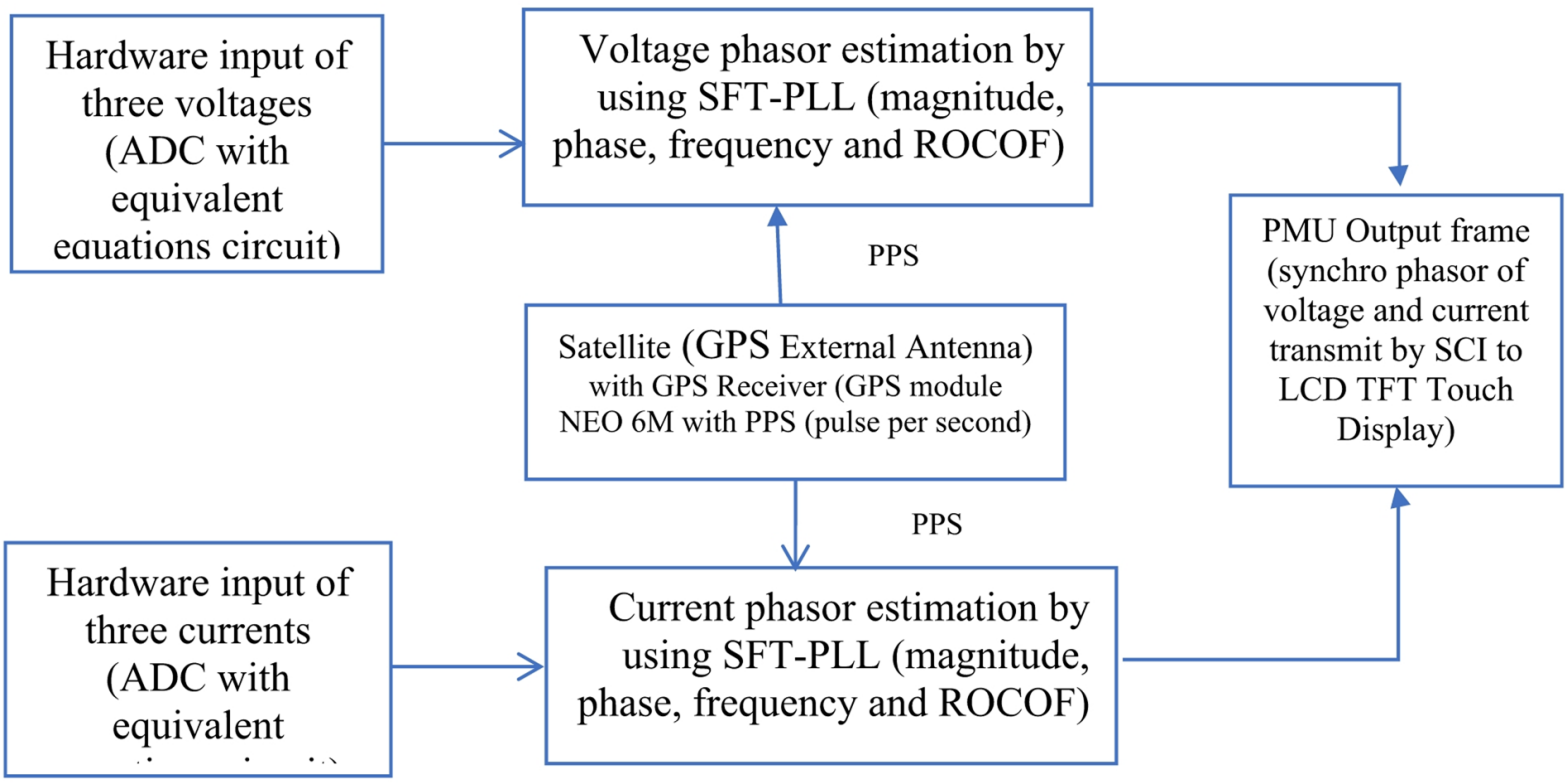
The block diagram of proposed PMU on the external mode of MATLAB Simulink.

Implements phasor estimation algorithms, typically using a Digital Signal Processor (DSP), in this work, Texas Instruments Delfino F28379D board is used. The proposed SFT-PLL method introduces an improved digital signal processing algorithm capable of accurately extracting voltage and current phasors even under distorted or noisy conditions, outperforming conventional techniques such as the Discrete Fourier Transform (DFT). The SFT-PLL technique has been implemented and the proposed PMU is designed in external mode of MATLAB Simulink. The PMU utilizes a 16-bit Analog-to-Digital Converter (ADC) integrated within the DSP for digitizing the input voltage and current signals. The 16-bit resolution allows the ADC to distinguish 65,535 discrete levels, providing high precision in representing the analog input signals in digital form. This resolution and simultaneous capturing of measured signals ensures accurate sampling of the three-phase voltage and current, which is critical for reliable phasor estimation and frequency calculation. The ADC operates at a sufficiently high sampling rate to capture the dynamic behavior of the power system while maintaining synchronization with the GPS-based pulses per second of the GPS Module (NEO 6 M with GPS External Antenna). The role of the GPS is to do the synchronization of the phasors with the global time so that the grid data concentrators can get a synchronized picture of the multiple PMUs distributed all over of the grid. Figure 4 shows the proposed PMU on the external mode of MATLAB Simulink.

External Mode in MATLAB/Simulink is a very helpful feature that lets you see what is happening inside your system while it is running on the real hardware. Instead of rebuilding the code every time you want to test something, you can monitor signals live and change parameters instantly from Simulink. This makes it much easier to tune a PI controller, and check if the sensors, controllers, or filters are working correctly. This feature saves a lot of time during testing. Because of this, External Mode is widely used when developing embedded systems, especially when working with real-time applications like PMUs, power converters, or motor control.

As illustrated in Fig. 4, the proposed SFT-PLL technique is applied to the three-phase voltage and current signals to estimate their phasors and system frequency. The system operates at a nominal grid frequency of 50 Hz with a 5 kHz sampling rate, corresponding to 100 samples per fundamental cycle. This configuration balances computational load and estimation accuracy, with phasor and frequency outputs reported every 0.2 m second. Phasor synchronization is achieved using the Pulse-Per-Second (PPS) signal provided by the GPS module, which serves as a precise hardware time reference to align the phasor computation cycles with the UTC second boundary. The GPS antenna ensures reliable satellite reception, enabling the module to output a stable 1-PPS signal along with NMEA time strings. In this implementation, the PPS signal is directly interfaced with the DSP to synchronize the computation and the reporting frames, while the NMEA data are used only for coarse UTC time-stamping. Although the ADC sampling clock operates independently at 5 kHz and is not directly disciplined, the periodic alignment of the first reporting frame with the PPS signal effectively constrains clock drift and prevents long-term timing error accumulation. This PPS-disciplined synchronization approach ensures accurate UTC-aligned time-tagging of the estimated voltage and current synchrophasors in compliance with IEC 60255-118-1 requirements. The system reports phasor frames at a rate of 50 frames per second (one frame every 20 ms). The first frame of each second is synchronized with the PPS pulse, while the subsequent frames are generated at fixed intervals using the DSP internal timer, enabling high-resolution real-time monitoring. After computation, the synchronized voltage and current phasors, along with frequency, are encapsulated into data frames and transmitted via the SCI interface of the TI Delfino F28379D platform. The output frames can be visualized on a TFT LCD touch display.

### PMU outputs communication

The measurement data generated by the proposed PMU are transmitted using the IEEE C37.118 standard frame format, which encapsulates the estimated voltage and current phasors together with the system frequency. After the phasor estimation algorithm completes its processing on the DSP, the computed phasors and frequency values are organized into a structured data frame that fully complies with the standard communication requirements. To ensure reliable and error-free transmission, the data frame includes dedicated start and end identifiers, allowing the receiving device to accurately detect the beginning and completion of each packet. In addition, a Cyclic Redundancy Check (CRC) field is appended to the frame. This CRC is calculated on the transmitter side and verified on the receiver side, providing a robust mechanism to detect any data corruption during transmission. The completed and protected frame is then transmitted through the Serial Communication Interface (SCI) of the F28379D. For real-time visualization, the data are forwarded to an Arduino DUE board, which serves as the interface controller for the TFT LCD touch display. The Arduino DUE receives the SCI frame, validates its integrity using the start/end markers and CRC, extracts the phasor magnitudes, phase angles, and frequency, and finally displays the processed information on the TFT screen in a clear and user-friendly format. This approach achieves a simple, low-cost, and reliable solution for PMU output visualization while ensuring secure and accurate data transmission between the processing unit and the display module.

## Proposed SFT-PLL technique

The SFT-PLL algorithm in paper^28^ has been implemented to estimate the frequency and the phasor of the three-phase voltage or current where these three phases are converted to low voltage compatible for Delfino board. The voltages are represented by a balanced set of three-phase sinusoidal voltage Vg with a frequency *f*g (Hz) and with a peak value A (Volt) is represented in Eq. (1) where θ_0_ is the initial phase shift (rad) of the voltage $\:{V}_{ga}$ thus three-phase voltage *V*_g_ is given by

1 $$\begin{aligned}{V}_{ga}&=A\:\mathrm{cos}(2\pi\:{f}_{g\:}\:t+{\theta\:}_{0})\\ \:{V}_{gb}&=A\:\mathrm{cos}(2\pi\:{f}_{g\:}\:t-2\pi\:/3+{\theta\:}_{0})\\{V}_{gc}&=A\:\mathrm{cos}(2\pi\:{f}_{g\:}\:t+2\pi\:/3+{\theta\:}_{0})\end{aligned}$$

The three voltages will be processed using two sets of balanced three-phase signals, *V*x and *V*y, which will operate at a predetermined nominal grid frequency *f*n (Hz), have a unity peak value, and have the same phase sequence as the grid voltage. The initial set of signals *V*x is represented by sine functions known as the sine or direct set as shown in Eq. (2) and the second set of voltages *V*y, known as the cosine or quadratic set, is determined by cosine functions is represent by Eq. (3):

2 $$\begin{aligned}{V}_{xa}&={sin}\left(2\pi\:\:{f}_{n}\:t\right)\\{V}_{xb}&={sin}(2\pi\:\:{f}_{n}\:t-2\pi\:/3)\\ {V}_{xc}&={sin}(2\pi\:\:{f}_{n}\:t+2\pi\:/3)\end{aligned}$$

3 $$\begin{aligned}{V}_{ya}&={cos}\left(2\pi\:\:{f}_{n}\:t\right)\\{V}_{yb}&={cos}(2\pi\:\:{f}_{n}\:t-2\pi\:/3)\\ {V}_{yc}&=\:\mathrm{cos}(2\pi\:{f}_{n\:}\:t+2\pi\:/3)\end{aligned}$$

to obtain the angle *θ*_0_, the Fourier transform necessitates doing the following integrations represent by Eqs. (4) and (5) where the subscript *i* = a, b, c and $\:{T}_{n}=\frac{1}{{f}_{n}}$. For any positive and non-zero value of *f*_n_

4 $$\:{x}_{i}=\frac{1}{{T}_{n}}\underset{t}{\overset{t+{T}_{n}}{\int\:}}{v}_{xi\:\:}.{v}_{gi\:}dt$$

5 $$\:{y}_{i}=\frac{1}{{T}_{n}}\underset{t}{\overset{t+{T}_{n}}{\int\:}}{v}_{yi\:\:}.{v}_{gi\:}dt$$

**Fig. 5:**
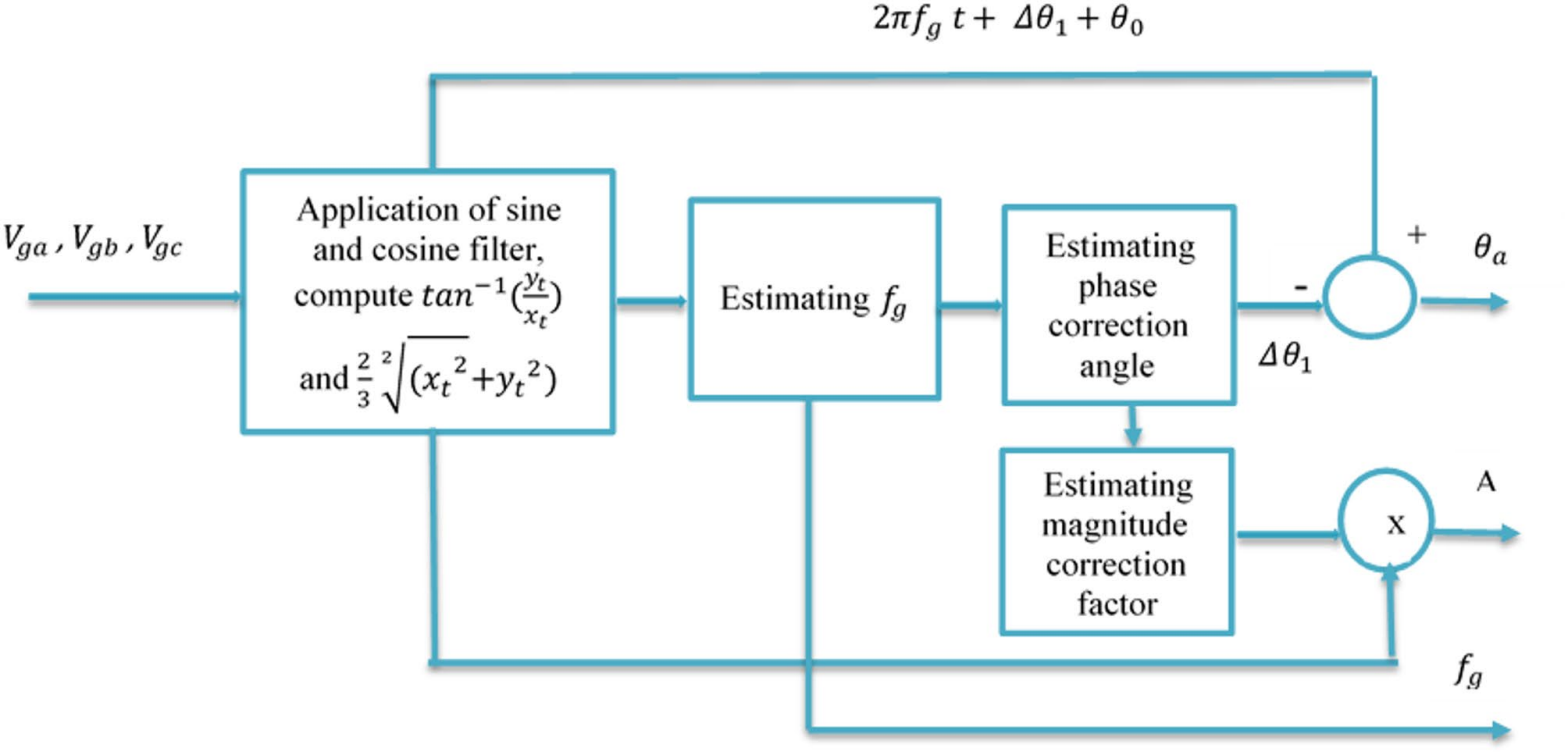
Schematic diagram of the proposed FT implementation for three-phase system.

Two cases are considered for evaluating these integrals. The first case corresponds to off-nominal operation, where ($\:{\boldsymbol{f}}_{\boldsymbol{g}\:}\ne\:{\boldsymbol{f}}_{\boldsymbol{n}}$), representing the general operating condition. The second case corresponds to nominal operation, where ($\:{f}_{g}={f}_{n\:}$), which can be regarded as a special case of the off-nominal condition. Figure 5 illustrates the complete implementation of the proposed Fourier Transform (FT)-based phasor estimation algorithm for a three-phase system. The process begins at the leftmost block, where the three-phase voltages are processed using sine and cosine filters operating at the nominal frequency $\:{f}_{n}$. The filtered components are summed to obtain the orthogonal quantities $\:{x}_{t}$ and $\:{y}_{t}$ as shown in Eqs. (6) and (7)

6 $$\:{x}_{t}=\sum\limits_{i=a}^{c}{x}_{i}=3{c}_{1}\mathrm{cos}({w}_{D}t+{\theta\:}_{0}+\varDelta\:{\theta\:}_{1})$$

7 $$\:{y}_{t}=\sum\limits_{i=a}^{c}{y}_{i}=3{c}_{1}\mathrm{sin}({w}_{D}t+{\theta\:}_{0}+\varDelta\:{\theta\:}_{1})$$

where

8 $$\:{w}_{D}=2\pi\:({f}_{g}-{f}_{n})$$

9 $$\:\varDelta\:{\theta\:}_{1}=\frac{{w}_{D}}{2{f}_{n}}$$

10 $$\:{c}_{1}=\frac{A\mathrm{sin}\varDelta\:{\theta\:}_{1}}{2\varDelta\:{\theta\:}_{1}}$$

from which the uncompensated phase angle is computed in the leftmost block as Eq. (11):

11 $$\:2\pi\:\left({f}_{g}-{f}_{n}\right)t+{\theta\:}_{0}+\varDelta\:{\theta\:}_{1}=\:{\mathrm{tan}}^{-1}\left(\frac{{y}_{t}}{{x}_{t}}\right)$$

Under off-nominal conditions, this phase contains a frequency-dependent deviation. In the intermediate block, the actual grid frequency $\:{f}_{g}\:$is estimated using Eq. (12)

12 $${f}_{g}=\frac{1}{2 \pi}.\frac{d}{dt}\left(\mathrm{tan}^{-1}\left(\frac{{y}_{t}}{{x}_{t}}\right)\right)+{f}_{n}$$

the phase angle correction term Δθ_1_ is calculated using the estimated $\:{f}_{g}$ in the next block. This term represents the phase error introduced due to the mismatch between the nominal filter frequency and the actual grid frequency. The true phase angle of the grid voltage $\:{V}_{ga}\:$is obtained by subtracting the correction term from the uncompensated phase angle as per Eq. (13) as follows.

13 $$\:{\theta\:}_{a}=\:{\mathrm{tan}}^{-1}\left(\frac{{y}_{t}}{{x}_{t}}\right)+2\pi\:{f}_{n}t-\frac{\pi\:({f}_{g}-{f}_{n})}{{f}_{n}}$$

Finally, the adjusted magnitude can be computed by simply calculating the magnitude correction factor from Eq. (14) in the bottom block.

14 $$\:A\:=\:\frac{2}{3}\cdot \:\frac{\varDelta\:{\theta\:}_{1}}{\mathrm{sin}\varDelta\:{\theta\:}_{1}} \cdot \left(\mathrm{tan}^{-1}\left(\frac{{y}_{t}}{{x}_{t}}\right)\right)+\sqrt[2]{\left({{x}_{t}}^{2}{{+y}_{t}}^{2}\right)}$$

Therefore, the outputs of the system are the corrected phase angle $\:{\theta\:}_{a}$ ,the compensated magnitude (A) and the estimated grid frequency $\:{f}_{g}$. When the system operates at nominal frequency ($\:{f}_{g}={f}_{n\:}$), the correction term Δθ1 approaches zero, and the algorithm operates in nominal mode. Under frequency deviation, the correction mechanism ensures accurate phase, frequency and magnitude estimation.

**Fig. 6:**
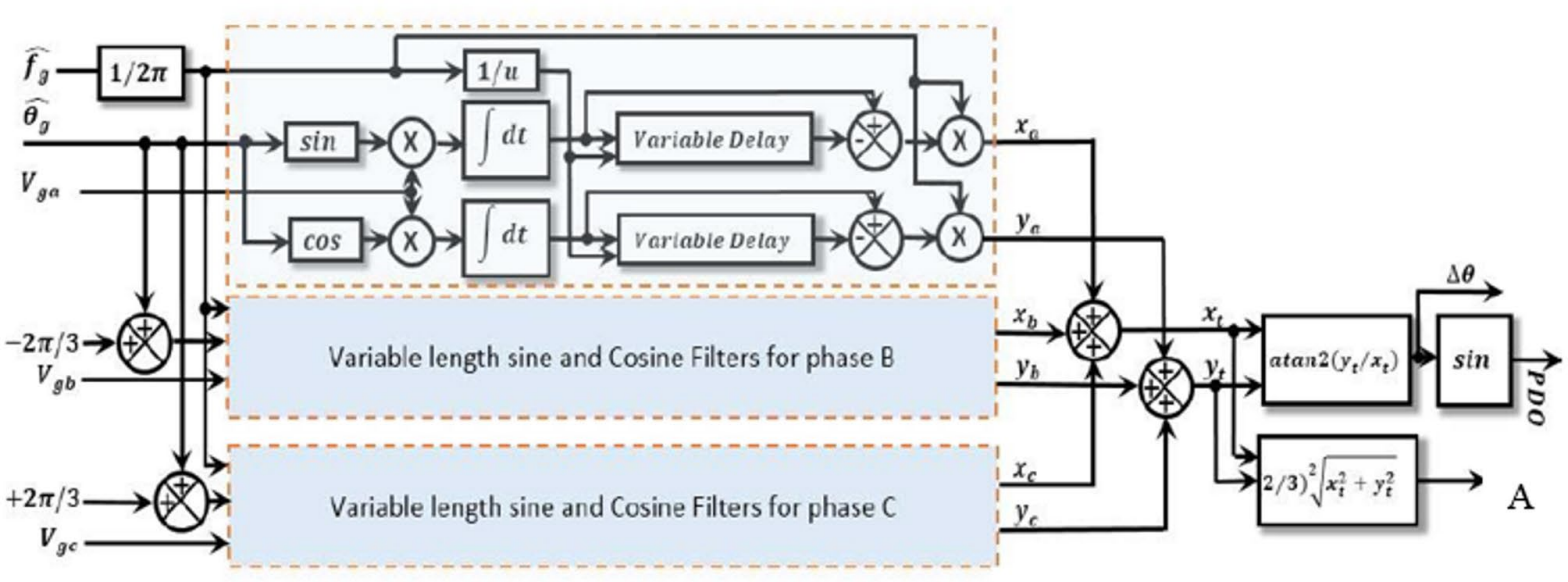
Schematic representation of the proposed variable window width SFT phase detector^28^.

**Fig. 7:**
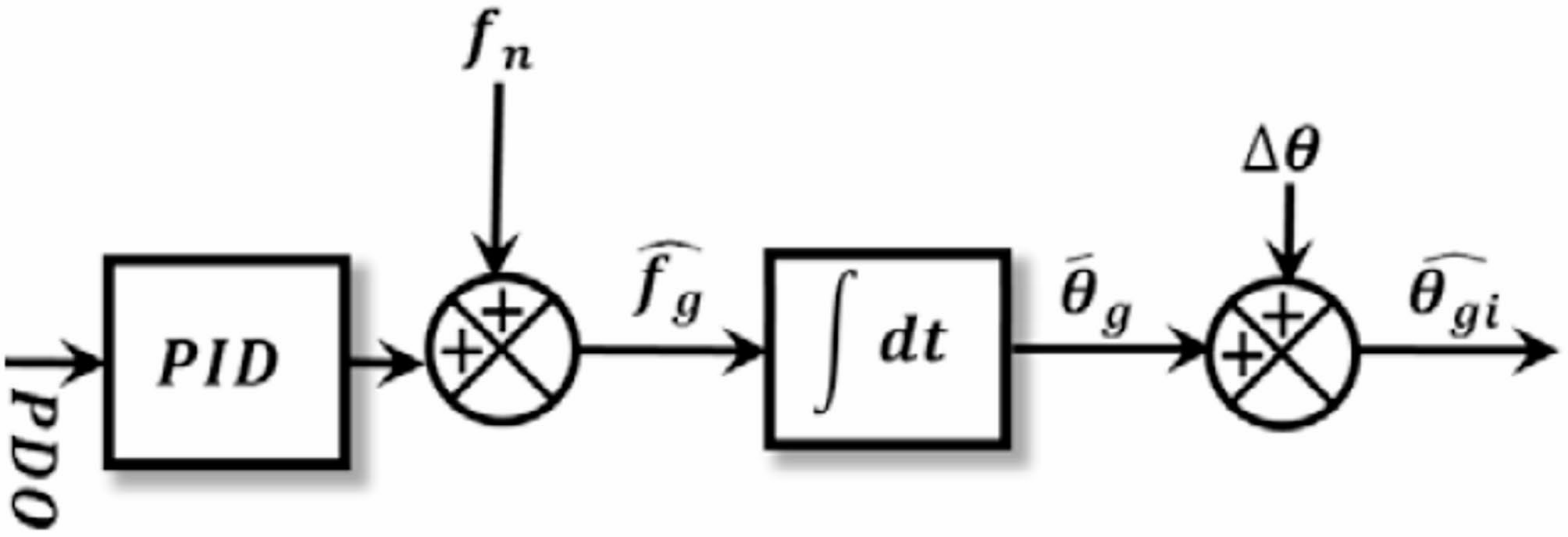
Phase-locked loop schematic representation^28^.

The schematic representation of this technique (SFT-PLL) is provided in Figs. 6 and 7. In Fig. 6, you can see that the shaded rectangles in phases b and c are constructed similarly to those in phase a.

Figure 7 illustrates the phase-locked loop (PLL) structure used to synchronize the Sliding Fourier Transform (SFT) with the actual grid frequency. The PLL aims to eliminate the phase difference Δθ between the internally generated reference signal and the phase extracted from the SFT.

The phase difference is defined as Eq. (15):

15 $$\:{\Delta\:}{\theta\:}\left(\mathrm{k}\right)={\theta}_{SFT}\left(\mathrm{k}\right)-{{\theta}_{\mathrm{g}}}^{\wedge}\left(\mathrm{k}\right)$$

where $\:{\theta}_{SFT}\left(\mathrm{k}\right)$ is the phase obtained from the Fourier block and $\:{{\theta}_{\mathrm{g}}}^{\wedge }\left(\mathrm{k}\right)$ is the estimated phase generated by the PLL. The phase-locked loop PID controller processes this phase difference Δɵ to produce a frequency correction signal by Eq. (16).

16 $$\:u\left(k\right)={K}_{P}{\Delta\:}{\uptheta\:}\left(k\right)+{K}_{i}\sum\:{\Delta\:}{\uptheta\:}\left(k\right)\Delta t+{K}_{d}\frac{{\Delta\:}{\uptheta\:}\left(k\right)-{\Delta\:}{\uptheta\:}\left(k-1\right)}{\Delta t}$$

The estimated grid frequency is then obtained as Eq. (17):

17 $$\:{{\mathrm{f}}_{\mathrm{g}}}^{ \wedge}\left(\mathrm{k}\right)\:={\:f}_{n}+u\left(k\right)$$

The corresponding angular frequency is by Eq. (18):

18 $$\:{{{\upomega\:}}_{\mathrm{g}}}^{ \wedge }\left(\mathrm{k}\right)={{2\:{\uppi\:}\:\mathrm{f}}_{\mathrm{g}}}^{ \wedge}{(k)}$$

The estimated phase is obtained by discrete-time integration is by Eq. (19)

19 $$\:{{\theta}_{\mathrm{g}}}^{\wedge }\left(\mathrm{k}\right)={{{\theta}_{\mathrm{g}}}^{\wedge }\left(\mathrm{k}-1\right){+\:{\upomega\:}}_{\mathrm{g}}}^{\wedge }\left(\mathrm{k}\right)\Delta t$$

To update the sine and cosine filters, the estimated frequency $\:\widehat{{f}_{g}}$ and the phase angle $\:\widehat{{\theta}_{g}}$ are fed back to the variable window SFT When the loop reaches the locked condition: Δθ disappears and the filter frequency $\:{f}_{n}$ changes to $\:{f}_{g}\:$. As a result, the PLL modifies the SFT’s frequency so that it operates at the real grid frequency when it locks. In this instance, the operation mode changes from off-nominal to nominal. This configuration preserves the renowned SFT strong harmonic rejection while enabling quick phase difference detection.

## Experimental results and validation of the proposed PMU

**Fig. 8:**
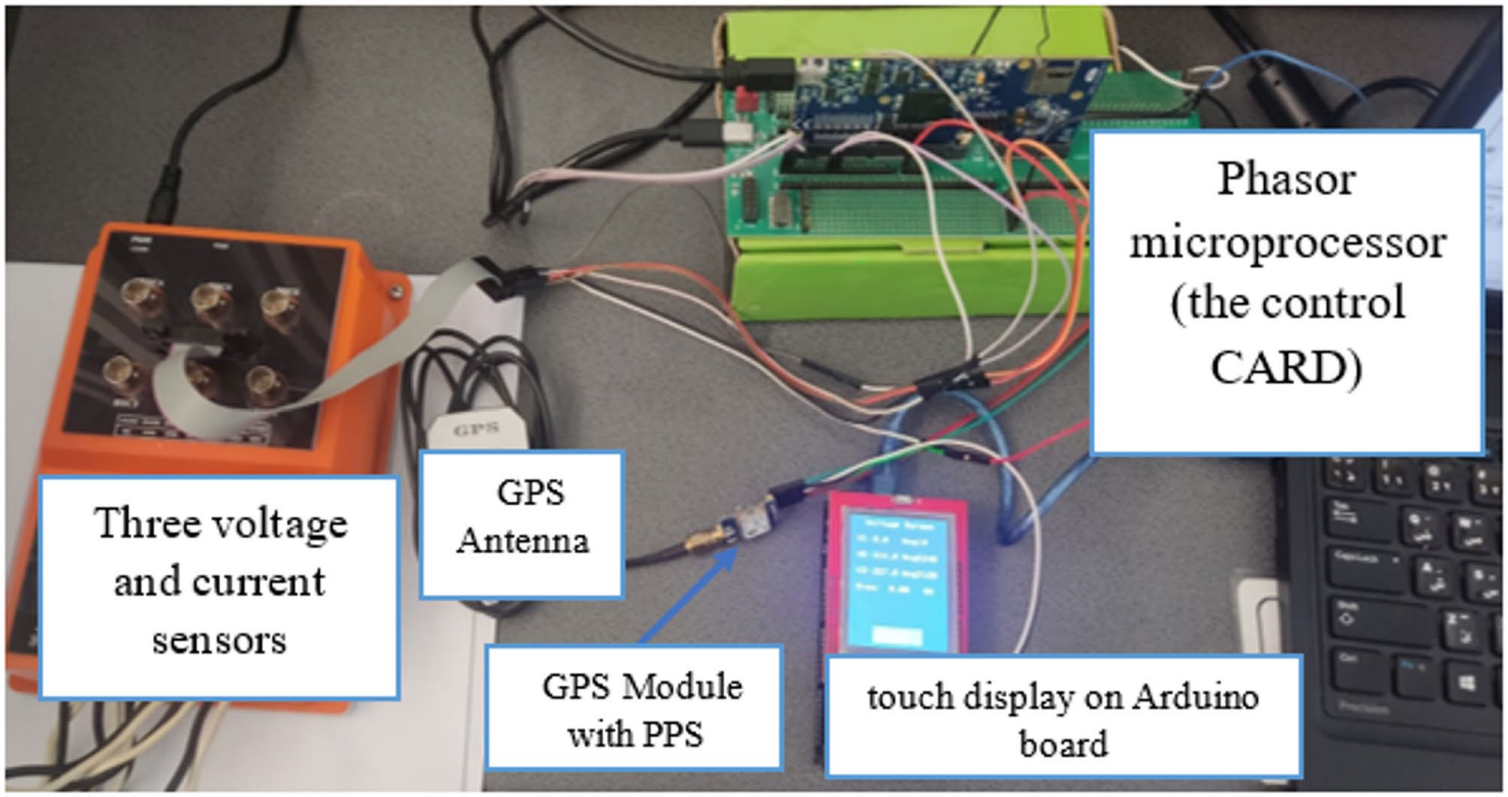
The hardware setup of the proposed PMU.

This section presents the experimental results obtained from the implementation of the proposed Phasor Measurement Unit (PMU) and evaluates its performance under different operating conditions. The aim is to verify the accuracy, reliability, and real-time operation of the PMU in estimating voltage and current phasors and frequency. The experimental setup includes the Delfino™ TMS320F28379D control CARD R1.3 board, the GPS-based time synchronization system, and the TFT LCD touch display for real-time visualization of the measured phasors as shown in Fig. 8.

The performance of the proposed PMU was experimentally validated on a real-time hardware prototype using three-phase voltage and current signals generated by the RX-33 Three-Phase Reference Standard. This setup provides precise and configurable reference signals, enabling accurate assessment of the PMU accuracy, reliability, and real-time performance under different operating conditions. Three-phase voltage and current were sourced to the primary side of the PEM sensors, which convert the high-voltage and high-current signals to levels suitable for the DSP input. Prior to testing, the sensors and the PMU input channels were carefully calibrated to ensure accurate measurement of instantaneous values. Once the system was energized, the PMU processed the input signals using the SFT-PLL algorithm and generated the corresponding voltage and current phasors along with the system frequency. The computed phasors and frequency values were displayed on the MATLAB scope as time-varying signals for monitoring and analysis of quality and speed of response with respect to grid events. This setup allowed for a comprehensive assessment of the PMU’s performance under controlled laboratory conditions.

To rigorously validate the proposed PMU prototype under these tests, its performance has been evaluated according to the IEC/IEEE 60255-118-1:2018^29,30^ standard with Total Vector Error (TVE) that is defined as Eq. (20).

20 $$\:TVE\left(N\right)=\frac{\sqrt{{({X}_{est}\left(n\right)-{X}_{ref}\left(n\right))}^{2}+{({Y}_{est}\left(n\right)-{Y}_{ref}\left(n\right))}^{2}}}{\sqrt{{\left({X}_{ref}\left(n\right)\right)}^{2}+{\left({Y}_{ref}\left(n\right)\right)}^{2}}}$$

where X and Y represent the real and imaginary components of the synchrophasor, respectively and n represent number of frame, Frequency Error (FE in Hz) is defined by Eq. (21),

21 $$\:FE={|f}_{est}\left(n\right)-{f}_{ref}\left(n\right)|$$

Rate of Change of Frequency (ROCOF) is defined by Eq. (22),

22 $$\:ROCOF\left(n\right)=\frac{{f}_{est}\left(n\right)-{f}_{est}\left(n-1\right)}{t}$$

with the $\Delta t$ = 20 msec. The equation of Rate of Change of Frequency Error (RFE in Hz/s) is defined in Eq. (23).

23 $$\:RFE={|ROCOF}_{est}\left(n\right)-{ROCOF}_{ref}\left(n\right)|$$

### Steady-state operating conditions (magnitude change)

#### For the three-phase voltage

**Fig. 9:**
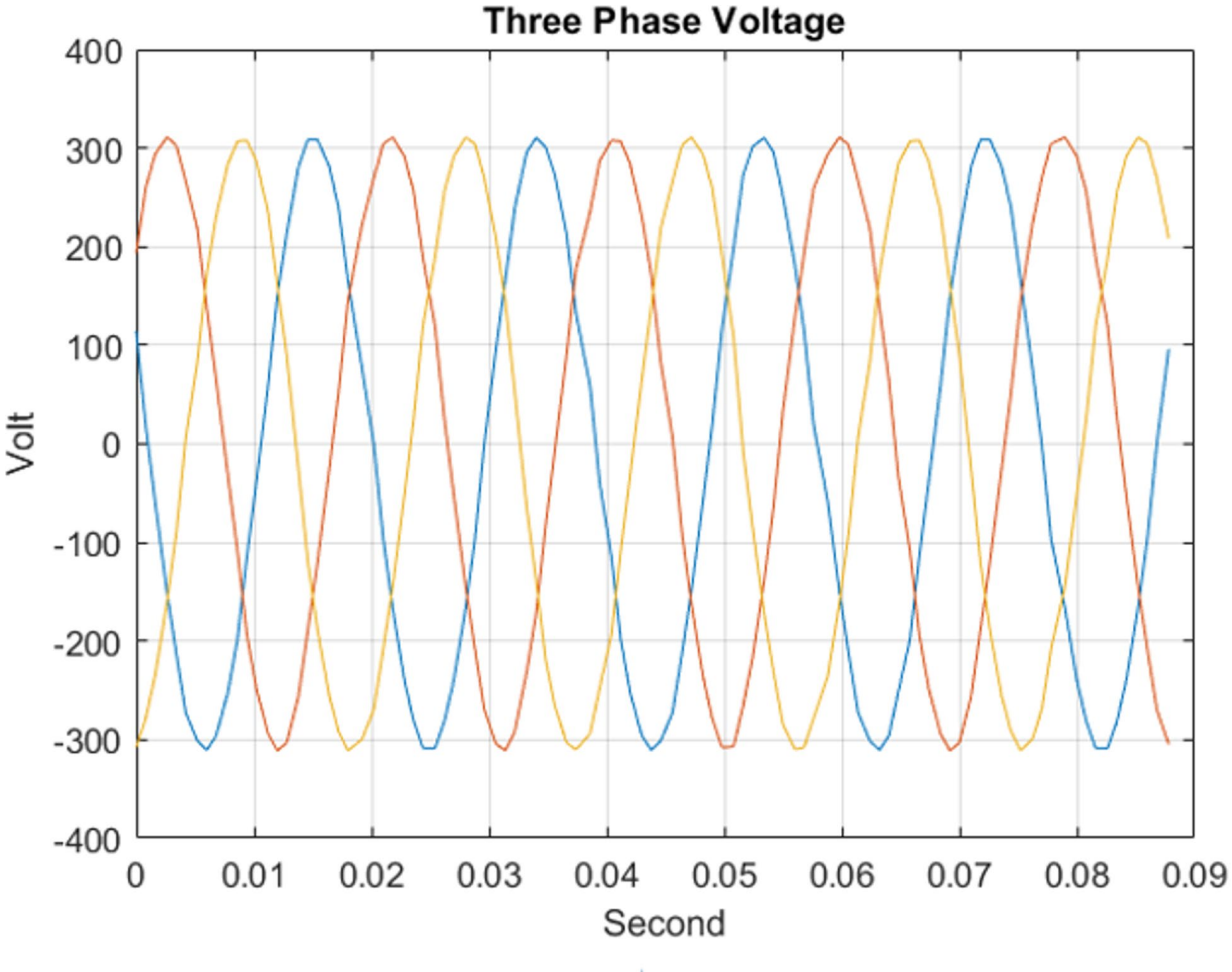
220 RMS volt three-phase voltage output of ADC of the Delfino™ TMS320F28379D board.

**Fig. 10:**
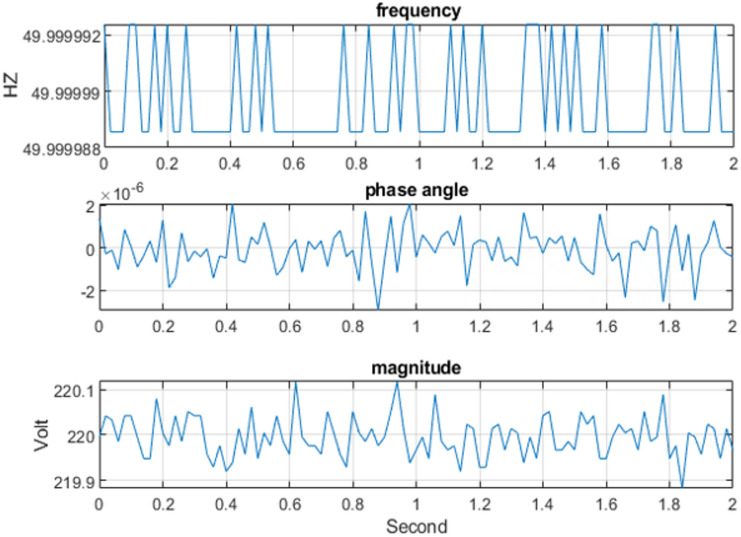
The output estimate frequency and the phasor of the voltage at 220 Vwith time per frame.

**Fig. 11:**
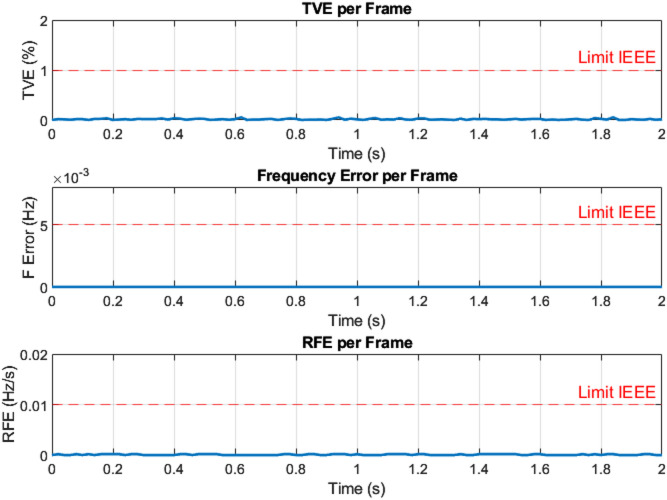
Total vector error (TVE), frequency error (FE) and rate of change of frequency error (RFE) per frame at 220 V.

The three-phase source was configured to supply voltages of 100 V, 200 V, 220 V, and 300 V at a frequency of 50 Hz under steady-state operating conditions for testing purposes. For example, the three-phase voltage at 220 V, as measured by the Delfino board with sampling time 0.0002 s, is presented in Fig. 9 versus time. The corresponding output, including the estimated voltage phasors and system frequency at 220 V per frame, is illustrated in Fig. 10. Figure 11 presents the Total Vector Error (TVE), Frequency Error (FE) and Rate of Change of Frequency Error (RFE) per frame at 220 V and as seen in the Table 2, the PMU demonstrates high accuracy across all tested voltage levels. It can be observed that the Total Vector Error (TVE), Frequency Error (FE), and Rate of Change of Frequency Error (RFE) remain very low across most operating conditions, indicating high measurement accuracy and stability where at all level the TVE is well below 1%, FE is below 0.005 Hz and RFE is below 0.01 Hz/s meeting the IEEE and IEC standard requirements^29,30^.

**Table 2:** The input voltage, maximum, mean and standard deviation of total vector error (TVE), frequency error (FE) and rate of change of frequency error (RFE) at different magnitude of voltage.

Actual input RMS voltage, F		Total vector error (TVE), frequency error (FE) and rate of change of frequency error (RFE)								
		Maximum			Mean			Standard deviation		
V	Hz	TVE %	EF (Hz)	RFE (Hz/s)	TVE %	EF (Hz)	RFE(Hz/s)	TVE %	EF (Hz)	RFE (Hz/s)
100.00	50	0.0643	1.53e^− 5^	3.81e^− 4^	0.0227	1.03e^− 5^	9.06e^− 5^	0.0155	1.81e^− 6^	9.95e^− 5^
200.00	50	0.0576	1.14e^− 5^	1.91e^− 4^	0.0172	1.00e^− 5^	9.06e^− 5^	0.0123	1.84e^− 6^	9.57e^− 5^
220.00	50	0.0537	1.14e^− 5^	1.91e^− 4^	0.0165	1.04e^− 5^	8.5e^− 5^	0.0119	1.71e^− 6^	9.53e^− 5^
300.00	50	0.1246	1.14e^− 5^	1.91e^− 4^	0.0461	8.72e^− 6^	6.04e^− 5^	0.0272	1.73e^− 6^	8.92e^− 5^

**Fig. 12:**
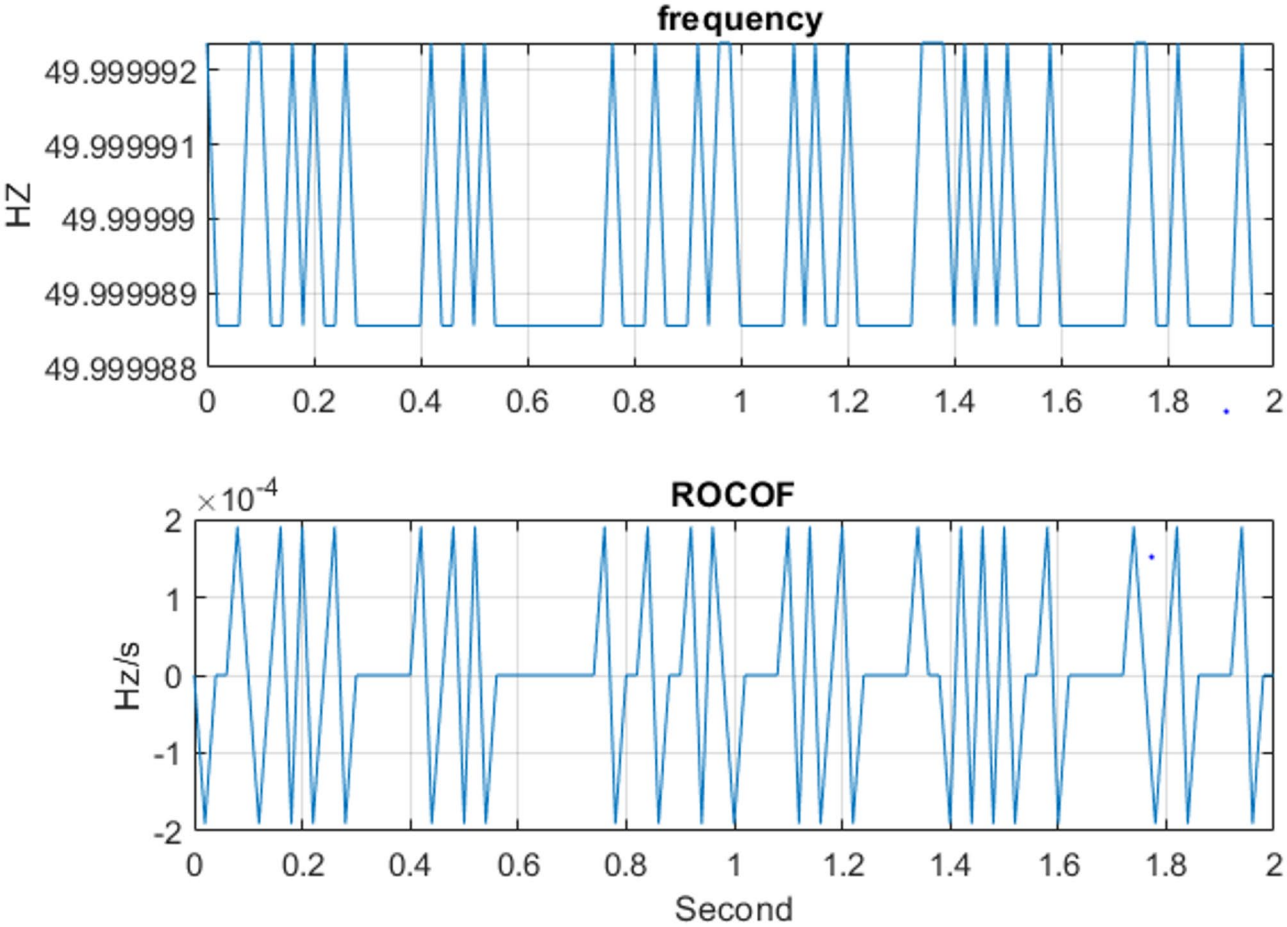
ROCOF and estimated frequency at 50 Hz for 220 V per frame.

As indicate in Fig. 12 the maximum ROCOF was 1.91e^-4^ Hz/s, while the mean absolute value was 8.5e^–5^ Hz/s, calculated per frame 0.02 s at 220 V with Eq. (22). these values correspond to a smooth frequency variation under steady-state operating conditions and full compliance with the IEC 60255-118-1 requirements for both Class P and Class M PMUs, which specify accuracy limits rather than magnitude constraints.

#### For the three-phase current

**Fig. 13:**
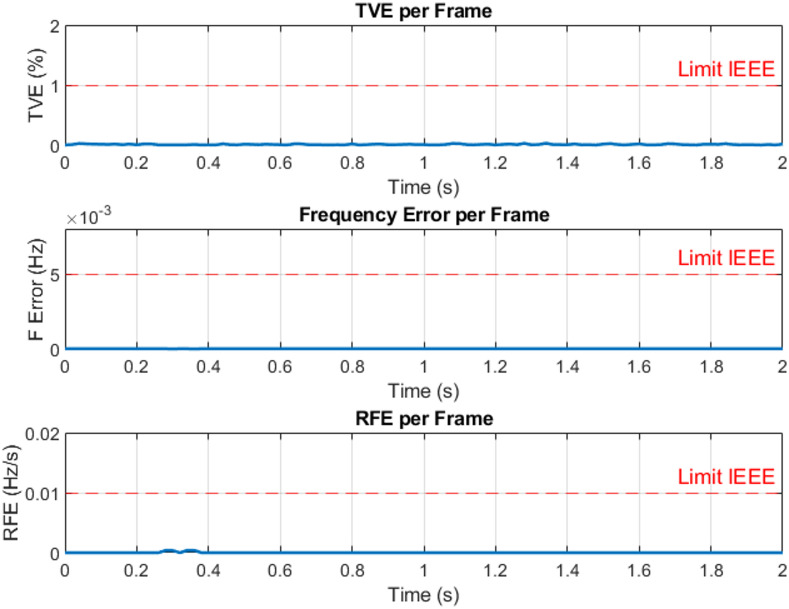
Total vector error (TVE), frequency error (FE) and rate of change of frequency error (RFE) per frame at 3 A.

**Fig. 14:**
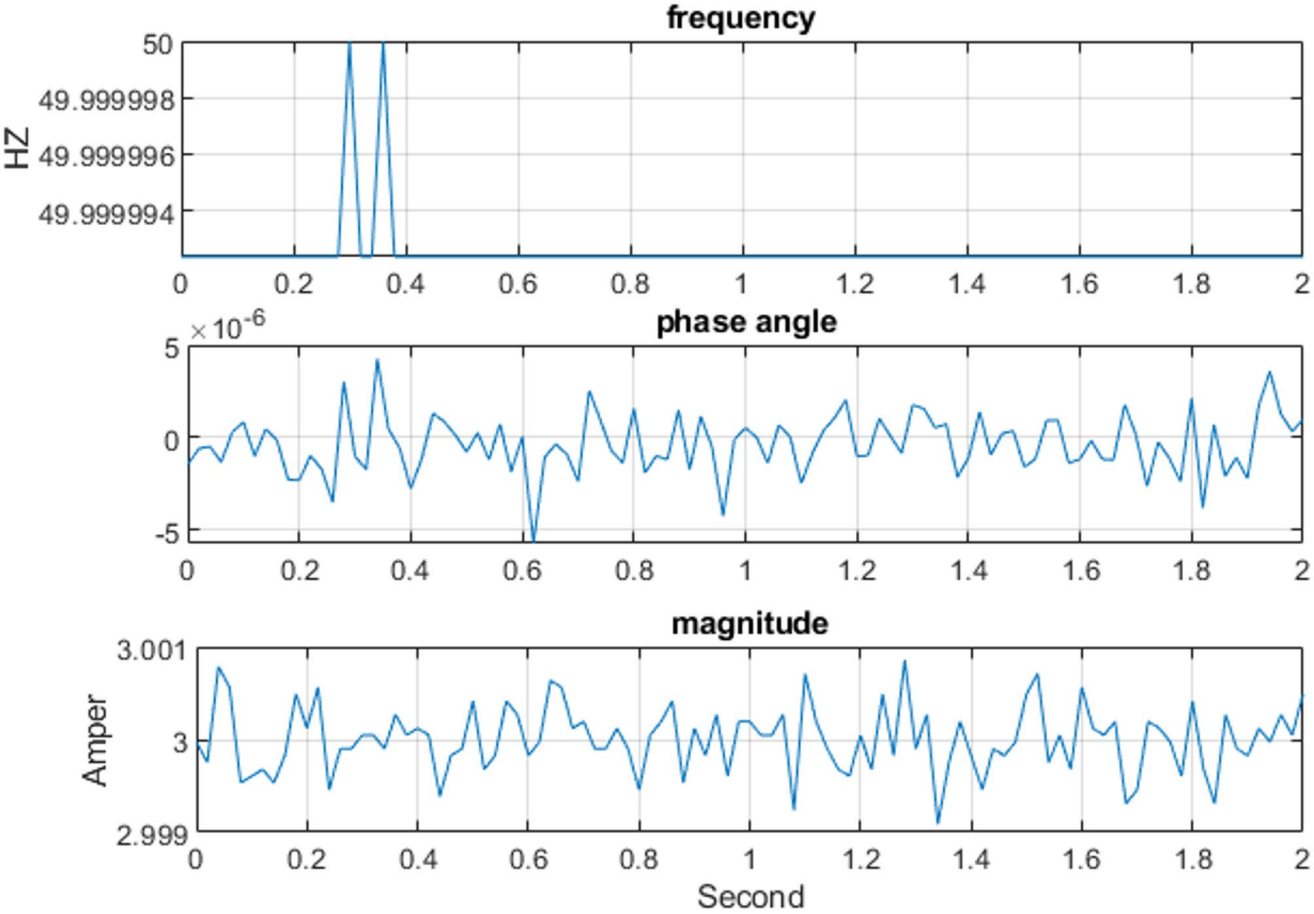
The output estimate frequency and the phasor of the current per frame at 3 A.

**Fig. 15:**
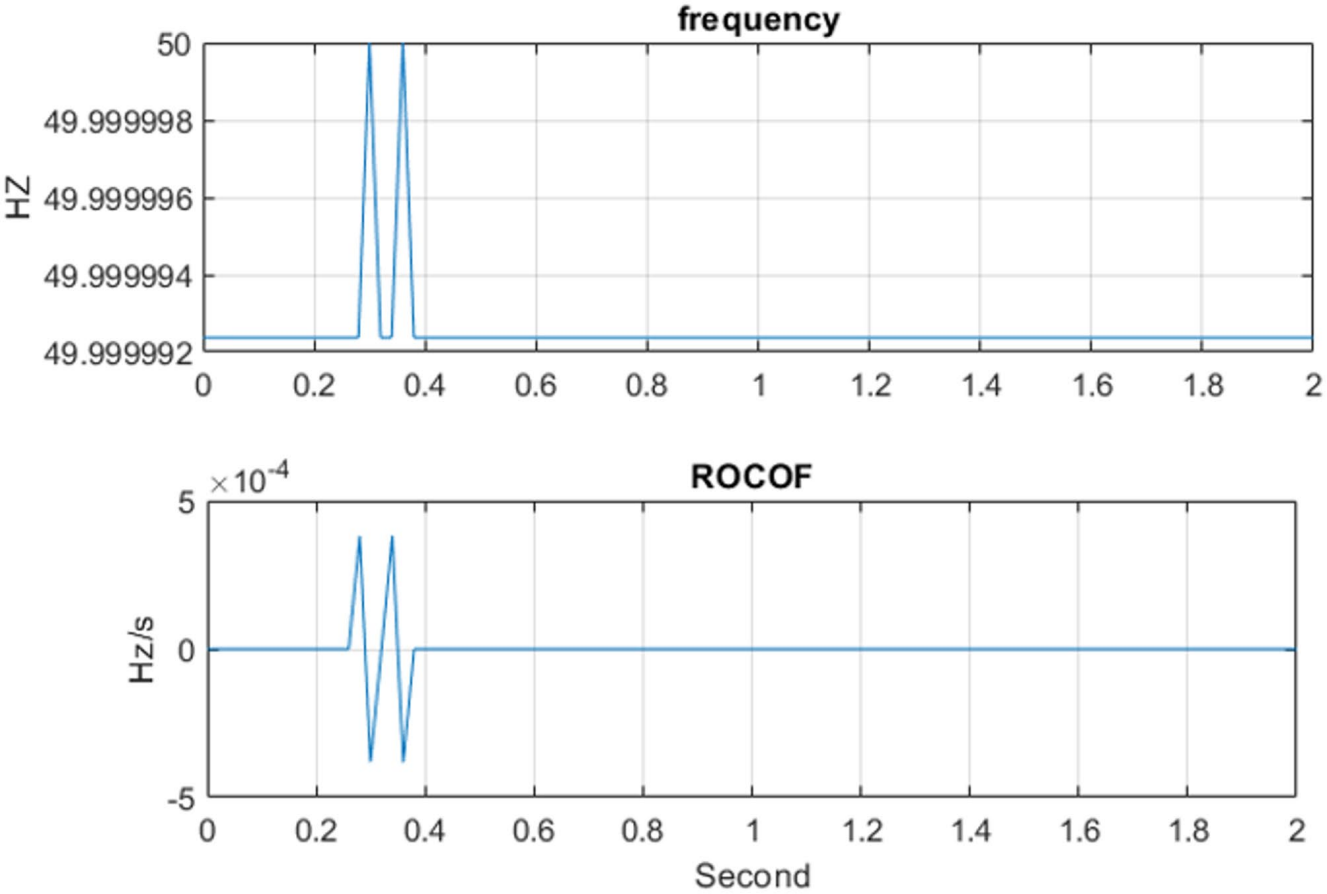
ROCOF and estimated frequency at 50 Hz per frame for 3 A.

The three-phase source was set to supply current levels of 1 A, 2 A, 3 A, 4 A, and 5 A at a nominal frequency of 50 Hz for testing. As an example, Fig. 13 illustrates the Total Vector Error (TVE), Frequency Error (FE), and Rate of Change of Frequency Error (RFE) per frame at a current level of 3 A. The corresponding phasor estimation results, including the extracted current phasors and the estimated system frequency at 3 A, are shown in Fig. 14, while Fig. 15 presents the ROCOF per frame under the same operating condition. As summarized in Table 3, the PMU demonstrates high measurement accuracy across all tested current levels. It is evident that the TVE, FE, and RFE remain very low under most operating conditions meeting the IEEE and IEC standard requirements^29,30^, reflecting both high accuracy and stability. Overall, these results confirm the reliability and effectiveness of the proposed PMU in accurately estimating both phasors and system frequency.

**Table 3:** The input current, maximum, mean and standard deviation of total vector error (TVE), frequency error (FE) and rate of change of frequency error (RFE) at different magnitude of current.

Actual input RMS current, F		Total vector error (TVE), frequency error (FE) and rate of change of frequency error (RFE)								
		Maximum			Mean			Standard deviation		
A	Hz	TVE %	EF(Hz)	RFE (Hz/s)	TVE %	EF(Hz)	RFE (Hz/s)	TVE %	EF(Hz)	RFE (Hz/s)
1.00	50	0.0512	7.63e^− 6^	3.81e^− 4^	0.0134	4.08e^− 6^	2.04e^− 4^	0.0116	3.82e^− 6^	1.91e^− 4^
2.00	50	0.0475	7.63e^− 6^	0	0.0161	7.63e^− 6^	0	0.0107	0	0
3.00	50	0.0303	7.63e^− 6^	3.81e^− 4^	0.0095	7.48e^− 6^	1.51e^− 5^	0.0073	1.07e^− 6^	7.48e^− 5^
4.00	50	0.0586	7.63e^− 6^	0	0.0154	7.63e^− 6^	0	0.0128	0	0
5.00	50	0.0858	7.63e^− 6^	1.91e-4	0.0265	7.63e^− 6^	7.93e-5	0.0208	0	9.45e-5

### Abnormal operating conditions

**Table 4:** Total vector error (TVE), frequency error (FE) and rate of change of frequency error (RFE) at different Abnormal operating conditions.

Test		Total vector error (TVE), frequency error (FE) and rate of change of frequency error (RFE)								
		Maximum			Mean			Standard deviation		
		TVE %	EF (Hz)	RFE (Hz/s)	TVE %	EF (Hz)	RFE (Hz/s)	TVE %	EF (Hz)	RFE (Hz/s)
Unbalance voltage		0.4465	4.2e^− 5^	3.81e^− 4^	0.3787	4.2e^− 5^	3.78e^− 6^	0.0277	0	3.8e^− 5^
Phase change (degree)	90	0.1330	7.63e^− 6^	3.81e^− 4^	0.0847	5.82e^− 6^	1.13e^− 4^	0.0169	3.26e^− 6^	1.75e^− 4^
	–90	0.1274	1.14e^− 5^	1.91e^− 4^	0.0732	9.4e^− 6^	8.12e-5	0.0157	1.91e^− 6^	9.48e^− 5^
	180	0.1600	7.63e^− 6^	5.72e^− 4^	0.1295	1.81e^− 6^	1.47e^− 4^	0.0088	3.22e^− 6^	1.87e^− 4^
	–180	0.1780	1.14e^− 5^	3.81e^− 4^	0.1296	8.65e^− 6^	7.55e^− 5^	0.0107	1.94e^− 6^	1.01e^− 4^
Harmonic test (order)	2	0.5705	2.21e^− 4^	0	0.5219	2.21e^− 4^	0	0.019	0	0
	3	0.0583	1.14e^− 5^	1.91e^− 4^	0.0173	9.1e^− 6^	8.31e^− 5^	0.0118	1.86e^− 6^	9.5e^− 5^
	5	0.5629	2.71e^− 4^	0	0.5159	2.71e^− 4^	0	0.0204	0	0
	7	0.7561	2.71e^− 4^	0	0.6997	2.71e^− 4^	0	0.0197	0	0
	9	0.0497	7.63e^− 6^	0	0.0197	7.63e^− 6^	0	0.0126	0	0
	10	0.7186	3.09e^− 4^	1.91e^− 4^	0.6655	3.08e^− 4^	8.31e^− 5^	0.0194	1.83e^− 6^	9.5e^5^
	3,5	0.6083	6.75e^− 4^	3.81e^− 4^	0.5505	6.74e^− 4^	1.28e^− 4^	0.0205	3.17e^− 4^	1.81e^− 4^
Unbalance with Harmonic	3,5,7	0.3752	0.0015	3.81e-4	0.3216	0.0015	8.31e^− 5^	0.0212	3.54e^− 6^	1.58e^− 4^

#### Unbalance test

**Fig. 16:**
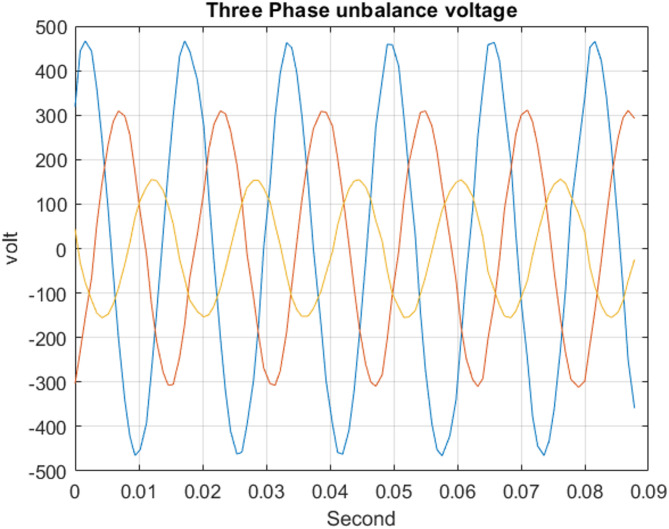
Unbalance three phase voltage output of ADC.

**Fig. 17:**
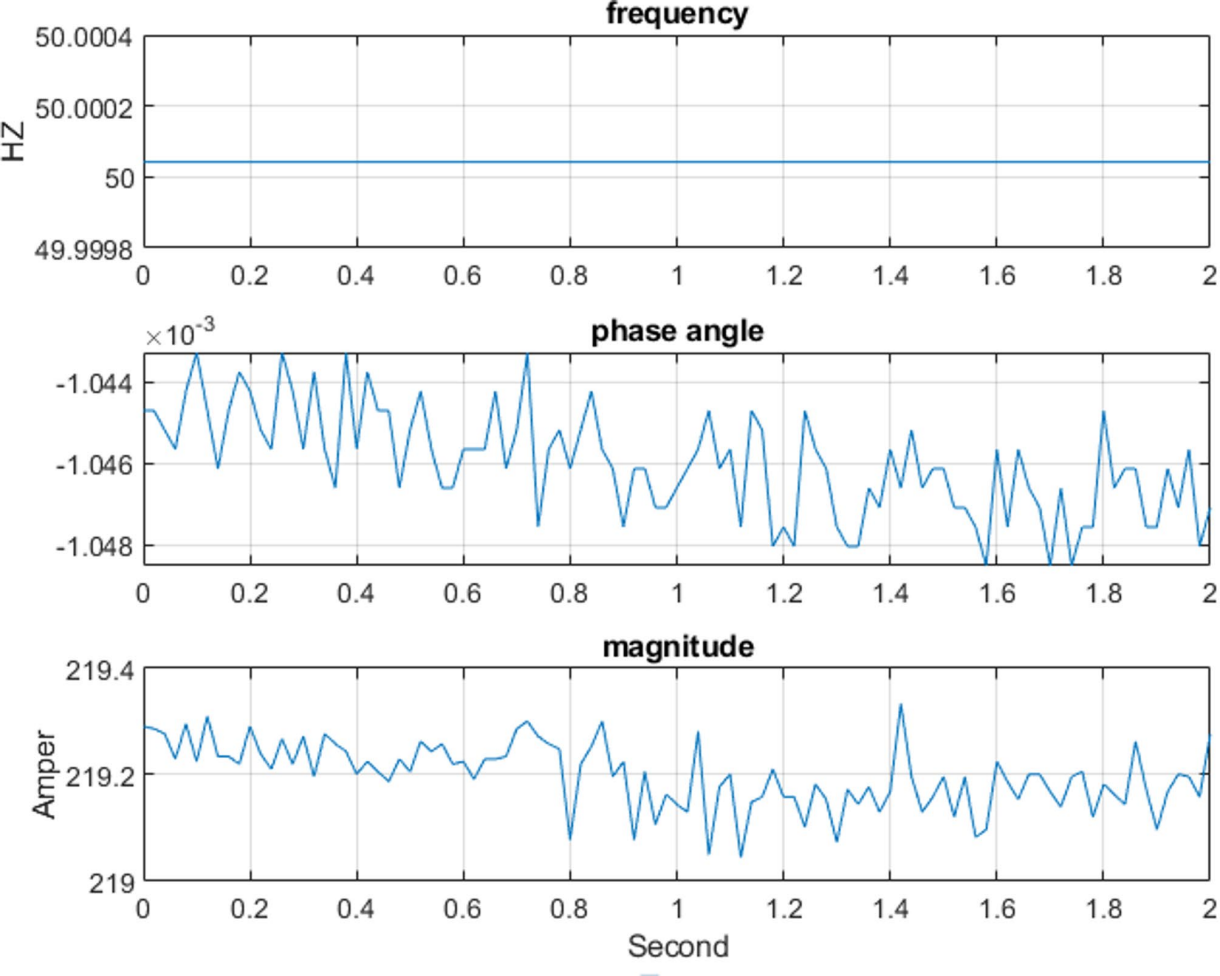
The output estimate frequency and the phasor of the voltage per frame at unbalance voltage.

**Fig. 18:**
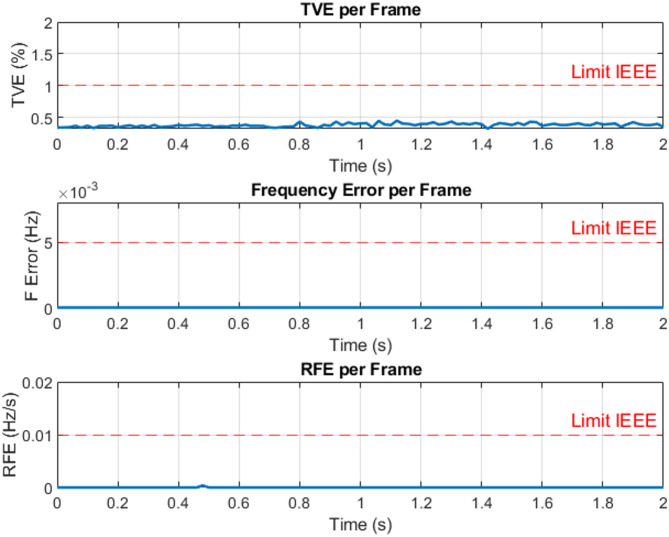
Total vector error (TVE), frequency error (FE) and rate of change of frequency error (RFE) per frame at unbalance voltage.

The unbalance test was performed by intentionally introducing a severe voltage unbalance among the three phases. The voltage of phase A was increased by 50% relative to the nominal value, while the voltage of phase C was decreased by 50%. Phase B was maintained at the nominal voltage of 220 V as shown in Fig. 16. This test scenario represents an extreme unbalanced operating condition and was applied to evaluate the robustness of the PMU frequency, and synchrophasor measurements under highly asymmetric voltage conditions. During this test, the measured maximum frequency error value of 4.2e^−5^Hz, while REF maximum is 3.81e^− 4^ and maximum TVE is 0.8373% as indicated in Table 4, The output estimate frequency and the phasor of the voltage per frame at unbalance voltage are shown in Fig. 17 and Total Vector Error (TVE), Frequency Error (FE) and Rate of Change of Frequency Error (RFE) per frame are present in Fig. 18, that indicating proper phase tracking and précised estimated frequency under severe voltage unbalance.

#### Phase change test

The phase change test was conducted on a three-phase voltage signal with a magnitude of 220 RMS volts at 50 Hz, using phase angles of 90°, − 90°, 180°, and − 180°. For each phase angle, the Total Vector Error (TVE), Frequency Error (FE), and Rate of Change of Frequency Error (RFE) were calculated. As shown in Table 4, the maximum values of TVE, FE, and RFE are very small and fall within the limits specified by IEEE and IEC standard requirements.

#### Harmonic distortion test

**Fig. 19:**
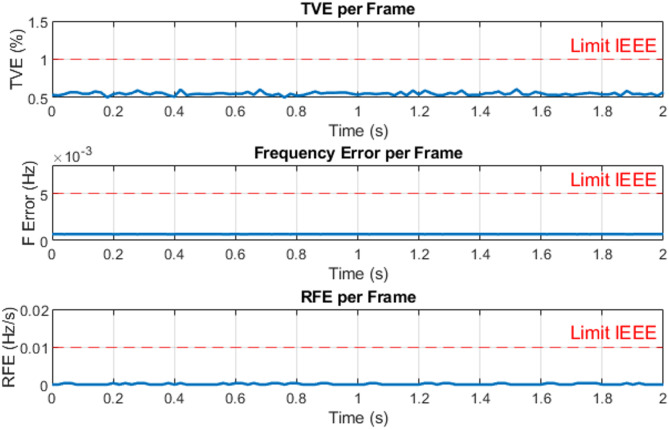
TVE, FE and RFE per frame at balance voltage with 3rd and 5th order harmonic.

**Fig. 20:**
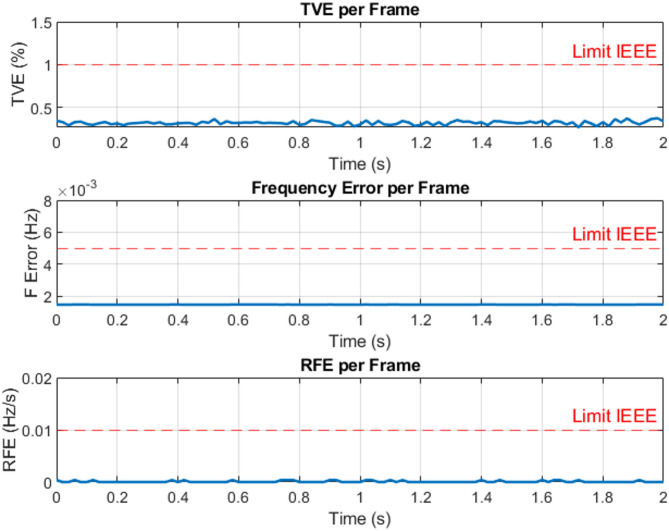
TVE, FE and RFE per frame at unbalance voltage with 3rd, 5th and 7th-order harmonic.

The harmonic performance of the proposed PMU was evaluated by injecting different harmonic components into a three-phase voltage signal with a nominal magnitude of 220 V. Individual harmonic tests were carried out at the 2nd, 3rd, 5th, 7th, 9th, and 10th orders, where each harmonic had a magnitude of 10% of the fundamental component. In addition, a combined harmonic scenario was tested by superimposing a 10% 3rd-order harmonic with a 5% 5th-order harmonic. A more challenging condition was also considered, where the system was subjected to both harmonic distortion and voltage unbalance. In this case, 10% of the 3rd harmonic, 5% of the 5th harmonic, and 3% of the 7th harmonic was added, while the three-phase voltages were made unbalanced such that phase A was increased by 50%, phase B remained at 220 V, and phase C was decreased by 50%. For all the aforementioned test scenarios, the Total Vector Error (TVE), Frequency Error (FE), and Rate of Change of Frequency Error (RFE) were calculated. The obtained results show that all performance indices remain within the limits specified by IEEE and IEC standard requirements, as summarized in Table 4; Figs. 19 and 20 show TVE, FE and RFE per frame for balance and unbalance voltage with harmonic distortion, confirming the high accuracy and robustness of the proposed PMU under both distorted and unbalanced operating conditions.

## Conclusion

In this paper, a highly accurate Phasor Measurement Unit (PMU) was successfully designed, implemented, and experimentally validated. The proposed PMU employs the SFT-PLL technique for precise estimation of voltage and current phasors, as well as system frequency, while GPS-based time synchronization ensures full alignment with the global UTC reference. It is of critical importance the resolution of the ADC converter (16-bit minimum resolution is required, beter accuracy can be granted by using higher resolution), and the exact synchronization of the capturing process. Comprehensive experimental testing was carried out under various nominal voltage levels (100 V, 200 V, 220 V, and 300 V at 50 Hz) and a wide range of current levels from 1 A up to 5 A. In addition to nominal steady-state conditions, the PMU was further evaluated under several abnormal operating scenarios, including phase change, voltage unbalance, and harmonic distortion. These tests were conducted to assess the robustness and reliability of the proposed phasor estimation technique under realistic grid disturbances. The experimental results confirm the excellent performance of the developed system: TVE for voltage and current measurement remained below 1%, across all tested levels, and frequency estimation errors did not exceed 0.005 Hz and RFE did not exceed 0.01 Hz/s, fully satisfying the IEC 60255-118-1 requirements. Overall, the proposed PMU provides a practical, accurate, and cost-effective solution for real-time phasor measurement in modern power systems. Its modular and open-architecture design, combined with its validated performance under frequency deviations, unbalanced conditions, and harmonic disturbances, makes it a strong candidate for smart grid integration, laboratory use, and research applications, offering a robust platform for monitoring, control, and future development.

## Data Availability

The data sets used and/or analyzed during the current study are available from the corresponding author on reasonable request.

## References

[1] Hojabri, M. & Dersch, U. Antonios papaemmanouil and peter bosshart a comprehensive survey on phasor measurement unit applications in distribution systems. *Energies.* **12**, 4552. 10.3390/en12234552 (2019).

[2] Cui, M., Wang, J., Tan, J., Florita, A. R. & Zhang, Y. A novel event detection method using PMU data with high precision. *IEEE Trans. Power Syst.* **34**(1), 454–466 (2019).

[3] Chitti Babu, B., Sridharan, K., Rosolowski, E. & Leonowicz, Z. Analysis of SDFT based phase detection system for grid synchronization of distributed generation systems. *Eng. Sci. Technol. Int. J.* **17**, 270–278 (2014).

[4] Luo, P. et al. A new measurement algorithm for PMU inpower system based on all-phase Fourier transform. *EURASIP J. Wirel. Commun. Netw.*10.1186/s13638-019-1492-3 (2019).

[5] Macii, D., Belega, D. & Petri, D. IpDFT-tuned estimation algorithms for PMUs: overview and performance comparison. *Appl. Sci.* **11**, 2318. 10.3390/app11052318 (2021).

[6] Bertocco, M., Frigo, G., Narduzzi, C., Muscas, C. & Pegoraro, P. A. Compressive sensing of a taylor-fourier multifrequency model for synchrophasor estimation. *IEEE Trans. Instrum. Measure.* **64**(12), 3274–3283 (2015).

[7] Zhan, L., Liu, Y. & Liu, Y. A clarke transformation-based DFT phasor and frequency algorithm for wide frequency range. *IEEE Trans. Smart Grid*. **9** (1), 67–77 (2018).

[8] de la Serna, A. & Rodriguez-Maldonado, J. Instantaneous oscillating phasor estimates with Taylor k-Kalman filters. *IEEE Trans. Power Syst.* **26**(4), 2336–2344 (2011).

[9] Roscoe, A. J., Abdulhadi, I. F. & Burt, G. M. P and M class phasor measurement unit algorithms using adaptive cascaded filters. *IEEE Trans. Power Delivery*. **28** (3), 1447–1459 (2013).

[10] de la Serna, J. A. Synchrophasor measurement with polynomial phase-locked-loop taylor–fourier filters. IEEE Trans. Instrum. Measure. **64**(2), 328–337 (2015).

[11] Huang, C., Xie, X. & Jiang, H. Dynamic phasor estimation through dstkf under transient conditions. *IEEE Trans. Instrum. Meas.* **66**(11), 2929–2936 (2017).

[12] Femine, A. D., Gallo, D., Landi, C. & Luiso, M. The design of a low cost phasor measurement unit. *Energies.* **12**, 2648. 10.3390/en12142648 (2019).

[13] Schofield, D., Mohapatra, D., Chamorro, H. R., Roldan-Fernandez, J. M., Abdellah, K. & Gonzalez-Longatt, F. Design and implementation of low-cost phasor measurement unit: phasorscatcher. *Energies.* **15**, 3172 (2022).

[14] Angioni, A., Lipari, G., Pau, M., Ponci, F. & Monti, A. A low cost PMU to monitor distribution grids. In Proceedings of the AMPS 2017—IEEE International Workshop on Applied Measurements for Power Systems, Liverpool, UK, 20–22 September (2017).

[15] Femine, A. D., Gallo, D., Landi, C. & Luiso, M. A design approach for a low cost phasor measurement unit. *2019 IEEE International Instrumentation and Measurement Technology Conference (I2MTC)*, Auckland, New Zealand, pp. 1–6, (2019). 10.1109/I2MTC.2019.8826889

[16] Du, L., Huang, J. & Liu, Q. A Realization of measurement unit for phasor measurement unit based on DSP. *2012 Asia-Pacific Power and Energy Engineering Conference*, Shanghai, China, pp. 1–3. 10.1109/APPEEC.2012.6307689 (2012).

[17] IEEE Std C37.118.1–2011; IEEE Standard for Synchrophasor Measurements for Power Systems. IEEE Power & Energy Society: Piscataway, NJ, USA (2011).

[18] Livanos, N. A. I. et al. OpenEdgePMU: an open PMU architecture with edge processing for future resilient smart grids. *Energies* **16**, 2756. 10.3390/en16062756 (2023).

[19] Garcia-Valle, R., Yang, G. Y., Martin, K. E., Nielsen, A. H. & Stergaard, J. DTU PMU laboratory development—testing and validation. In Proceedings of the IEEE PES Innovative Smart Grid Technologies Conference Europe, ISGT Europe, Gothenburg, Sweden, 11–13 October ; pp. 1–6. (2010).

[20] Sampson, O. V. *Construction of a Phasor Measurement Unit (PMU) for Power System Applications*. Ph.D. Thesis, University of Manitoba, Winnipeg, MB, Canada, (2015).

[21] Mohapatra, D. *Development and Hardware Implementation of a Phasor Measurement Unit Using Microcontroller*. NIT Rourkela. Available online: March 2022. (2015).

[22] Laverty, D. M. et al. The open PMU platform for open-source phasor measurements. *IEEE Trans. Instrum. Measure.* **62**(4), (2013).

[23] Laverty, D. M., John Morrow, D., McKinley, A. & Cregan, M. Open PMU: open source platform for synchrophasor applications and research. *IEEE Trans. Instrum. Measure.* (2011).

[24] Open Source SynchroPhasor PMU-CodePlex archive. Available online: https://archive.codeplex.com/?p=gridtrak (accessed on 4 Sep 2018).

[25] Kumar, M. & Kumar, M. S. Performance evaluation of M-class synchrophasor estimation using enhanced band-pass FIR filter under a comprehensive integrated compliance test. *Electr. Eng.* **107**, 4063–4075. 10.1007/s00202-024-02667-3 (2025).

[26] Rana, A. S., et al. Design and implementation of low-cost PMU for off-nominal frequency and DDC in compliance with IEEE C37.118 standard. *Distrib. Generation Altern. Energy J.* **38_2**, 519–546. 10.13052/dgaej2156-3306.3827 (2023).

[27] De Carvalho, G. U., Denardin, G. W., Cardoso, R. & Grando, F. L. A PID SRF-PLL based algorithm for positive-sequence synchrophasor measurements. *Int. Trans. Electr. Energy Syst.* **31**, e12777 (2021).

[28] Arafa, O. M., Abdallah, M. E. & Aziz, G. A. A. Frequency adaptive sliding fourier transform for synchronizing VSI to the grid. *Int. J. Power Electron. Drive Syst* **10**(2088–8694), 1034 –1048 (2019).

[29] IEEE/IEC International Standard-Measuring Relays and Protection Equipment-Part 118-1: Synchrophasor for Power Systems-Measurements; (IEEE: Piscataway, NJ, USA, 2018).

[30] IEEE. IEEE Standard for Synchrophasor Measurements for Power Systems–Amendment 1: Modification of Selected Performance Requirements; IEEE Std C37.118.1a-2014 (Amendment to IEEE Std C37.118.1–2011); (IEEE: Piscataway; pp. 1–25, NJ, USA 2014)

